# Integrating Sensor Technologies with Conversational AI: Enhancing Context-Sensitive Interaction Through Real-Time Data Fusion

**DOI:** 10.3390/s25010249

**Published:** 2025-01-04

**Authors:** Joseph C. Kush

**Affiliations:** Department of Instruction and Leadership, Duquesne University, Pittsburgh, PA 15282, USA; kushjoe@gmail.com

**Keywords:** artificial intelligence, ChatGPT, conversational AI, sensor technology

## Abstract

This article examines how sensor technologies (such as environmental sensors, biometric sensors, and IoT devices) intersect with conversational AI models like ChatGPT 4.0. In particular, this article explores how data from different sensors in real time can improve AI models’ comprehension of surroundings, user contexts, and physical conditions. Lastly, this article delves into the scientific principles supporting sensor technologies, data processing methods, and their fusion with generative models such as ChatGPT to develop adaptable, dynamic systems that engage with humans intelligently in real time. Some of the specific topics that are investigated include the science behind sensor networks and acquiring real-time data, how ChatGPT can analyze sensor data to generate dialogue that is sensitive to context, instances in healthcare (such as using wearable sensors along with AI chatbots for patient treatment), and smart homes (interaction with AI assistants driven by sensors). These subjects will prove advantageous for researchers in sensor technology as well as AI development, showcasing interdisciplinary progress in smart systems.

## 1. Introduction

The introduction of sensor networks, Internet of Things (IoT) devices, and real-time data collection systems has brought about significant changes in industries such as healthcare, environmental monitoring, and industrial automation [1,2]. These IoT-enabled networks connect physical devices and sensors, creating a web of interconnected systems that allow for constant monitoring of key metrics and enable the development of solutions that can quickly adjust to changing conditions based on context [3]. The integration of IoT technology has enhanced the ability to collect, share, and analyze vast amounts of data in real time, forming the backbone of smart systems that respond dynamically to environmental and situational changes.

The core of these advancements lies in the ability to quickly obtain, understand, and communicate data, a capability increasingly powered by the integration of artificial intelligence (AI) for complex decision-making processes. The Internet of Things (IoT) amplifies these capabilities by delivering diverse, real-time data streams that enhance AI’s contextual awareness and adaptability. This seamless interaction between IoT devices and AI not only highlights the transformative potential of these technologies but also underscores the essential role of the sensor industry in enabling this innovation.

By integrating live IoT data with AI-powered platforms like ChatGPT, advanced comprehension of data and timely, interactive responses are now achievable. For instance, sensors embedded in IoT devices can continuously monitor and relay information about environmental conditions, user behaviors, or system statuses. These data enable ChatGPT to adapt dynamically to changing contexts, offering personalized, meaningful interactions across applications.

The combination of the IoT and AI represents a groundbreaking step forward in industries such as healthcare, smart homes, and industrial automation, where responsive and intelligent systems are crucial. These advancements further cement the sensor industry’s pivotal role in fostering smarter, more adaptive environments and creating opportunities for innovation that will shape the future of connected systems.

This article examines how sensor technologies (e.g., environmental sensors, biometric sensors, IoT devices) can be effectively integrated with conversational AI models (such as ChatGPT) to improve these models’ comprehension of their surroundings, user contexts, and physical conditions for real-time, intelligent human interactions. The synergy between the IoT and conversational AI presents a transformative potential for creating adaptive, smart systems that engage dynamically with humans in various applications.

### 1.1. Fundamentals of Sensor Networks

Sensor networks are made up of geographically dispersed devices that can track a range of biological, chemical, and physical data [4,5]. These networks frequently consist of inexpensive, tiny sensors that can be widely used to gather large datasets. Every sensor in the network is usually made to identify particular biological or environmental factors, allowing for focused reactions and real-time insights [6]. Depending on the architecture of the network and the speed needed for data analysis, the acquired data are subsequently processed by either a central system or a distributed processing unit. These networks’ sensor types are quite diverse and suited to various applications:

- Environmental sensors: These sensors measure factors such as temperature, humidity, pressure, and air quality and are frequently used in applications ranging from environmental monitoring to smart home systems [7,8].
- Biometric sensors: These devices monitor physiological parameters like heart rate, respiration, and body temperature, making them integral to healthcare and fitness applications [9].
- Motion sensors: Employed in security systems, robotics, and smart homes, motion sensors detect movement within a designated area, providing immediate data on activity or potential intrusions [10].
- Chemical sensors: Designed to detect specific gases, liquids, or chemical substances, these sensors are crucial in industrial environments, where they monitor safety conditions, as well as in environmental monitoring, where they track pollutant levels [11,12].
- Acoustic sensors: Acoustic sensors capture sound waves or vibrations, finding applications in surveillance, industrial monitoring, and healthcare, particularly in the detection of physiological sounds such as heart and lung auscultations [13].

Every type of sensor is crucial in its field, providing precise, instantaneous data that improve the system’s awareness and allow quick reactions to environmental changes or potential dangers [14]. Yet, combining these various sensor types into a unified network poses difficulties related to data processing, system compatibility, and the requirement for strong algorithms to handle and assess extensive datasets.

### 1.2. Design of Sensor Networks

Sensor networks are typically structured using a multi-tier design, which is essential for facilitating the efficient collection, transmission, and analysis of data [14]. This hierarchical approach allows data to flow seamlessly from the lowest level, where the data are generated, to higher levels, where the data can be aggregated, analyzed, and acted upon.

At the foundational tier, sensor nodes are equipped with several critical components to enable their functionality. These components include the following:

- Sensing elements: Devices such as temperature sensors, accelerometers, or biometric detectors, which capture specific physical or environmental parameters.
- Processing units: Typically microcontrollers or microprocessors that handle basic computations, data filtering, and initial analysis directly on the sensor node.
- Communication hardware: Modules such as Wi-Fi, Bluetooth, Zigbee, or LoRa that enable data transmission between nodes or to centralized systems.
- Power sources: Often batteries or energy-harvesting mechanisms, such as solar panels, to sustain long-term operation in remote or resource-limited environments.

To optimize energy usage and minimize bandwidth consumption, sensor nodes often implement localized data processing. By performing tasks such as data filtering, aggregation, and compression at the node level, the system reduces the volume of data transmitted to higher tiers [15,16]. This approach not only extends the operational lifespan of the network by conserving energy but also improves the overall efficiency.

A key technological advancement supporting this architecture is edge computing, also known as edge processing. In this paradigm, computational tasks are performed closer to the data source—often directly on or near the sensor nodes—rather than relying solely on centralized servers. Edge computing has gained widespread popularity in sensor networks due to its ability to do the following:

- Reduce latency: By processing data locally, it eliminates delays associated with transmitting data to distant servers for analysis.
- Enhance system reliability: Distributed processing ensures that even if the central system is unreachable, critical computations can continue at the edge.
- Alleviate centralized system load: By handling preliminary analysis and filtering at the edge, it minimizes the burden on central systems, freeing them for more complex tasks.

This integration of edge computing with sensor networks not only enhances performance but also enables real-time applications in diverse fields such as healthcare, environmental monitoring, and industrial automation [17]. The synergy between localized processing and efficient communication design ensures that sensor networks can meet the demands of increasingly complex and dynamic operational environments.

The transmission of data across the network is made easier by the communication infrastructure in the middle layer. The layer can be set up with either wired or wireless connections, but wireless communication protocols such as Wi-Fi, Bluetooth, ZigBee, and LoRa are popular due to their affordability, minimal power usage, and ability to grow in size [18,19]. Wireless protocols provide flexibility for establishing networks in settings where wiring may be impractical, expanding deployment possibilities.

The top level consists of sites that aggregate data, commonly referred to as “sink nodes”. These nodes collect data from multiple sensors for extra processing or decision-making purposes [5]. Aggregation points occasionally employ advanced data processing tools like AI or machine learning algorithms to examine sensor data and provide immediate actionable responses [2]. Sensor networks can expand efficiently due to their hierarchical structure, which also enhances data processing and energy usage throughout the system.

### 1.3. Wireless Sensor Networks (WSNs)

Wireless sensor networks (WSNs) are a type of sensor network that allows for remote monitoring and control where wired infrastructure is not possible or is costly [20]. WSNs use low-power, short-range communication technologies like ZigBee, LoRa and Bluetooth Low Energy (BLE) to ensure a long battery life and reliable communication [21,22]. These are important when sensor nodes need to operate for extended periods without frequent battery replacements or recharging. WSNs can cover large areas, forming dense networks that provide wide coverage for activities like environment monitoring and urban infrastructure management [7]. WSNs in smart cities monitor traffic, pollution and energy consumption to provide real-time data for efficient resource allocation and quick responses to changing situations [23]. WSNs have proven their importance in relation to making decisions in dynamic, large-scale environments where real-time data are key.

## 2. Real-Time Data Acquisition and Processing

One of the key characteristics of contemporary sensor networks is their capacity to gather and analyze data instantaneously. Real-time data acquisition involves gathering data constantly as events happen, with no interruptions, enabling systems to immediately react to fluctuations in the environment or situations.

### 2.1. Data Acquisition in Sensor Networks

In sensor networks, data acquisition is achieved by sampling environmental or physiological variables in a structured way to capture changes in real time or near real time. The sampling strategies vary depending on the application. Some systems sample continuously, others use event-based or threshold-triggered sampling to manage the data volume and power [15,24]. For example, in environmental monitoring, sensors might sample variables like air quality or temperature to observe small changes over time [8]. In contrast, healthcare applications use biometric sensors that only activate when significant changes like heart rate or respiratory function changes suggest a health issue [9,25].

Selecting the correct sampling rate is crucial when designing sensor networks. Increased sampling frequencies offer more precise information but also result in a larger amount of data that must be sent, analyzed, and saved, potentially placing a burden on resources [26]. This issue is especially urgent in wireless sensor networks (WSNs), where it is crucial to save battery power [27]. Maintaining network performance and sustainability relies on striking a perfect balance between data resolution and transmission efficiency [18,28]. Adaptive sampling methods, which modify the sampling frequency based on data patterns or surrounding conditions, have proven to be very successful in handling the data flow and saving energy while maintaining data accuracy [29].

### 2.2. Real-Time Data Processing

After data are collected in sensor networks, the data are processed to produce insights. For real-time systems, processing has to happen as data are being collected to support fast decision-making—a critical need in industries like industrial automation, autonomous vehicles and healthcare [6]. The key data processing techniques in these networks are edge computing, data fusion and machine learning. Edge computing allows data to be processed where the data are generated, usually in sensor nodes or adjacent edge devices. This minimizes latency and saves bandwidth by processing data at the edge of the network, so you do not have to transfer large data volumes to cloud servers [17,30]. That is why edge computing is particularly useful in time-critical scenarios like industrial automation and autonomous vehicles, where even milliseconds can impact safety and efficiency [31]. In smart manufacturing, edge computing allows machines to make adjustments quickly using real-time sensor data, increasing efficiency and reducing delays [32].

Data fusion combines information from several sensors to offer a more comprehensive view of the environment, enhancing decision-making through improved precision and reliability [33]. Autonomous vehicles use cameras, LiDAR, and radar sensors together to generate a precise 3D map of the surroundings, enabling precise navigation and effective avoidance of obstacles. Likewise, the integration of data from different sensor types in environmental monitoring improves understanding of complex ecosystems [34]. Sensor networks are increasingly incorporating machine learning algorithms to process and analyze real-time data. Machine learning excels at identifying patterns, detecting anomalies, and making predictions based on historical and current data [35]. Within an industrial setting, machine learning algorithms can predict equipment failures through analysis of sensor data patterns, allowing for proactive maintenance and reducing costly downtime [36]. In smart cities, machine learning can analyze information from traffic sensors to enhance traffic flow and reduce congestion as conditions change [37].

## 3. Difficulties in Sensor Networks and Acquisition of Real-Time Data

Despite the benefits offered by sensor networks and real-time data acquisition in various fields, it is still very important to address several key challenges in order to ensure security, efficiency, and ease of integration. The most critical challenges relate to the aspects of data protection, energy consumption, and scalability. The introduction of sensor networks in sensitive areas such as healthcare and smart cities raises some critical concerns about the protection of privacy and data. Biometric or geolocation data are two examples of sensitive, personal and sometimes classified information that sensor networks deal with, which enjoy some degree of confidentiality from non-authorized persons [38,39]. In this regard, the use of TLS or SSL protocols in combination with appropriate encryption practices is considered critical for maintaining data integrity and confidentiality while the information is being transferred across networks [40]. As a consequence, the data that sensor networks collect pose a greater challenge to the security of the whole system thanks to the volume of information collected. Risk can be reduced by the use of multiple security layers, such as access control, authentication and intrusion detection; however, these have to be balanced against performance and latency requirements [41].

Energy efficiency is a critical issue in sensor networks, particularly in wireless networks, where sensor nodes are typically battery-powered. Addressing this challenge is not only vital for the functionality of these systems but also pivotal for advancing the broader sensor industry. As the demand for sensor networks grows in diverse fields such as healthcare, environmental monitoring, and industrial automation, the industry is increasingly driven to innovate energy-efficient solutions that ensure reliability and scalability. Maintaining low energy usage while preserving data quality and frequency remains a fundamental design hurdle [42]. However, this challenge also represents an opportunity for sensor manufacturers and developers to lead in creating cutting-edge technologies that address these limitations. Approaches like duty cycling, which involves intermittently disabling sensors when they are not actively collecting data, have shown significant promise in prolonging battery life [14].

Additionally, by adopting low-energy communication protocols such as Zigbee, LoRa, and Bluetooth Low Energy (BLE), sensor networks can operate more efficiently while minimizing power consumption. Incorporating energy-harvesting techniques—such as solar power, vibration energy, and thermal energy harvesting—further reduces the reliance on conventional battery sources. These advancements not only improve the sustainability of sensor networks but also open up new markets for eco-friendly sensor technologies [43,44]. The continuous development of energy-efficient solutions positions the sensor industry as a critical player in driving innovation toward sustainable and scalable network systems. With these advancements, sensor technologies are becoming integral to the global push for smarter, greener, and more adaptive infrastructure.

As sensor networks expand, scalability issues become critical. Managing and processing data from thousands or even millions of sensors in real time requires highly scalable network infrastructure and data management techniques [27]. Efficient network protocols, data aggregation strategies, and distributed processing frameworks are essential to maintain system performance as networks grow. For instance, data aggregation minimizes redundant transmissions by combining similar data from multiple sensors, thereby reducing the communication overhead and extending the network’s life span [5]. Distributed processing also enables data to be analyzed locally at the edge, reducing the burden on centralized systems and improving response times [30].

Today’s advanced technologies, such as autonomous vehicles, healthcare, and environmental monitoring, rely heavily on sensor networks and real-time data collection systems. These systems allow for ongoing collection and processing of data, establishing a strong base for advanced AI models that can interact dynamically with users, resulting in more intelligent and responsive systems. Nevertheless, addressing concerns like data privacy, power efficiency, and scalability is essential to fully capitalize on the capabilities of sensor networks. Continuous improvements in sensor and data processing technologies are predicted to address these obstacles, paving the way for more advanced and adjustable applications.

## 4. ChatGPT’s Ability to Process Sensor Data for Context-Sensitive Dialogue Generation

Developing adaptive dialogue systems that are aware of context is a major step forward in AI technology, as demonstrated by models such as OpenAI’s ChatGPT. In contrast to conventional conversational agents using static inputs such as text queries or predetermined prompts, ChatGPT can utilize live sensor data to go beyond scripted responses and provide customized, contextually intelligent interactions. This skill is particularly beneficial in sectors like healthcare, home automation, and customer service, where immediate sensor information provides crucial understanding of surroundings, user actions, and even well-being.

## 5. Comprehending the Generation of Dialogue That Is Sensitive to Context

Context-aware dialogue generation involves a conversational agent’s capability to modify its answers by considering details about the present circumstances, surroundings, or user. This differs from traditional dialogue systems, which provide set answers to particular questions, without considering the overall context of the conversation. In a healthcare environment, a virtual health assistant’s conversation could be enhanced by having access to the patient’s heart rate, temperature, and stress levels. Likewise, in a smart home situation, the system could modify its reactions depending on environmental elements like the room temperature, lighting conditions, or user’s presence.

The significance of context in conversational systems.

Different types of context can be grouped in various categories within dialogue systems:

- User context: Details regarding the user’s identity, likes/dislikes, previous engagements, mood, or physical well-being. For example, in the healthcare industry, a virtual assistant could analyze information from wearable devices to assess the individual’s health condition and modify its responses accordingly.
- Environmental context: Environmental Context refers to details related to the surroundings, such as the location, climate, moisture, and sound intensity. An intelligent home assistant could adjust its dialogue based on the time of day, altering recommendations for lighting or heating as necessary.
- Temporal context: This pertains to the specific time of day or other time-related elements. In a virtual assistant, responses like “Good morning!” or reminders about appointments, exercise, or mealtimes can be influenced by the time of day.

Incorporating these contextual layers into ChatGPT’s dialogue generation system could enhance interactions with greater depth and significance. The difficulty lies in making sure the system can handle various sensor inputs in real time and produce precise and pertinent responses.

## 6. How ChatGPT Analyzes Sensor Data for Dialogue That Is Sensitive to Context

As a language model that generates text, ChatGPT mainly learns from large amounts of text and predicts the upcoming word or phrase based on the input it is given. However, when real-time sensor data are included in the conversation process, the model needs to understand non-textual inputs like numerical readings or sensor data and effectively incorporate them with its language skills. The process of integration requires several important steps.

### 6.1. Data Integration and Preprocessing

To generate context-sensitive dialogue using real-time sensor data, preprocessing is crucial. Sensor data, usually in raw numerical form (e.g., temperature, heart rate, or light levels), need to be normalized, embedded, and structured in a way that ChatGPT can process and utilize within its input pipeline, enabling the model to make effective use of these data for generating natural language responses [35,45]. This process includes normalizing sensor values, creating contextual embeddings, and fusing multimodal data to support intelligent, context-aware interactions (Table 1).

**Explanation of Steps:**

1. **Start:** The process begins.
2. **Acquire Raw Sensor Data:** This is the initial step of obtaining the data from the various sensors (e.g., temperature sensor, heart rate monitor). The data are typically in raw numerical form.
3. **Data Preprocessing Needed?:** This decision point checks if raw data need to be processed, where the answer will most likely be a yes.
4. **Normalize Sensor Values:** If the answer is yes, the numerical data from the sensors are scaled and standardized. This brings the values to a common range, preventing certain sensor data from dominating others due to larger numerical values, and is important for consistent model input.
5. **Create Contextual Embeddings:** The normalized sensor data are transformed into vector representations (embeddings) that capture the contextual meaning. This allows the model to process the sensor data and its relationships to other contexts (like time).
6. **Structure Data for ChatGPT Input:** The contextual embeddings and processed sensor data are formatted in a way that is suitable for use as input for the ChatGPT model. This often involves converting the data into a specific structure or format expected by the model’s input pipeline.
7. **Fuse with Other Context:** Other contextual data, such as user history, location, etc., can be added and incorporated into one combined context representation.
8. **Input Data to ChatGPT:** The combined structured sensor and contextual data are then fed as input to the ChatGPT model.
9. **Generate Context-Aware Dialogue:** The ChatGPT model uses the processed sensor data, in conjunction with other contexts, to generate relevant and context-aware dialogue.
10. **End:** The process concludes with the generated dialogue.

Bringing sensor data to a standard range of values and formats is essential through normalization in order to align with the model’s expected input parameters. Temperature readings can vary between 0 and 100 °C, with heart rate measurements usually falling between 40 and 180 bpm. Methods such as min–max scaling are often employed to normalize these values, ensuring they are uniform and interchangeable among varying categories and measurements, thereby preserving the reliability of the data flow [46]. Normalization not only guarantees consistent data but also improves the model’s accuracy in interpreting inputs, facilitating smooth integration of data from different sensors and sources.

A key part of processing sensor data for AI-driven dialogue systems is transforming it into contextual embeddings. This involves converting numerical or categorical sensor data into high-dimensional vectors that preserve the relationships between different data points. This allows the model to better understand the nuances in environmental, physiological, or behavioral information [47]. For instance, a temperature reading of 22 °C could be encoded into an embedding that represents “comfortable room temperature” based on the past context, enabling ChatGPT to respond in a way that aligns with this understanding.

Sensor-driven dialogue systems frequently depend on information from different origins, such as environmental, physiological, and behavioral sensors, necessitating advanced techniques for merging data. Methods such as multi-layer neural networks and attention mechanisms assist the model in efficiently merging and ranking various data streams, enabling it to customize responses according to the context and significance of each input [48,49]. In healthcare environments, fusion techniques enable the model to combine vital-sign data with environmental factors like temperature or light, producing more accurate responses that depict the user’s physiological condition [50].

### 6.2. Contextual Dialogue Generation

After the sensor data are injected as input into the model, the next step is to generate context-aware responses. ChatGPT leverages a transformer-based architecture that works well in applications requiring context attention throughout long segments of text. With these methods, the system becomes capable of perceiving and responding to unique user situations at runtime, which enhances the relevance and personal connection of interactions in applicational domains like healthcare and smart home setups [48,51]. The self-attention mechanism is fundamental to ChatGPT’s capacity for nuanced, context-aware dialogue. Self-attention enables the model to assign variable importance to different elements within its input—whether linguistic components or external sensor data points. For example, in a healthcare application, a high heart rate reading from a wearable sensor might lead the model to prioritize responses that convey calmness or concern, depending on the situation. By dynamically weighting relevant data, self-attention mechanisms allow ChatGPT to tailor its responses to align with the real-time physiological state of the user, offering contextually relevant interactions [48,52].

In addition to adapting content, ChatGPT can adjust its tone and level of formality according to contextual sensor inputs. In a smart home setting, when a motion sensor identifies the user in a particular room, ChatGPT can offer tailored suggestions like dimming the lights or adjusting the thermostat, enabling a smooth, interactive space [53]. This ability to respond appropriately to different situations improves the user experience by enabling the model to mimic human-like attentiveness and demonstrate awareness of the context in real time [54,55].

### 6.3. Example Applications of Sensor-Driven Context-Sensitive Dialogue

Combining sensor information with ChatGPT has the potential to revolutionize the capabilities of AI-powered chatbots across multiple industries, positioning the sensor industry as a critical enabler of next-generation smart systems. In the field of healthcare, for example, wearable sensors and IoT devices can continuously monitor a patient’s key health indicators, such as the heart rate, blood pressure, and oxygen levels. These advancements showcase the transformative role of sensors in improving patient care and underscore the industry’s importance in driving innovation. These data can be seamlessly transferred to a virtual health aide powered by ChatGPT, enabling the chatbot to deliver highly personalized, context-aware interactions. For instance, if a smartwatch detects an elevated heart rate, ChatGPT could respond with: “I noticed that your heart rate is higher than usual. Are you feeling okay? Would you like me to suggest a relaxation exercise or remind you to take a break?” Such personalized and timely responses are made possible by real-time sensor data, which add depth, significance, and relevance to AI-driven conversations. The integration of advanced sensor technologies with conversational AI not only enhances the user experience but also demonstrates the growing importance of the sensor industry in creating dynamic, adaptive systems. As industries increasingly adopt sensor-driven AI applications, the demand for innovative and high-quality sensor solutions is expected to rise, further cementing the industry’s role as a cornerstone of technological progress.

ChatGPT can adapt its conversations in smart home settings by utilizing sensor information like temperature, humidity, and occupancy. This results in a smoother user experience. For instance, when the temperature rises in a room and a motion sensor picks up a user’s presence, ChatGPT might say, “It’s starting to feel a little toasty in this space”. “Do you want me to change the temperature on the thermostat?”. This reply is customized for the particular situation, taking into account the current status of the environment and providing a personalized, effective method for engaging with smart home systems.

In environmental monitoring applications, ChatGPT can utilize sensor data from various sources, like air quality sensors and weather stations, to provide users with interactive and conversational insights. For instance, in a smart city, a ChatGPT-driven assistant could be asked by a user, “What is the air quality like today?”. Utilizing live information from sensors in the vicinity, the assistant could report, “The current air quality in your location is considered ‘good’, with a PM2.5 measurement of 12 µg/m^3^. Do you want me to provide you with updates during the day?”. This method enables users to obtain current, context-specific information through a conversational layout.

## 7. Challenges in Processing Sensor Data for Dialogue Generation

Integrating sensor information into ChatGPT’s dialogue generation is a very good move; however, this development is overwhelmed by quite a number of both technical and practical obstacles. Naturally, sensor data are volatile and subject to fluctuations, which can cause inaccuracy. Therefore, it is a great disadvantage to AI models. This problem is most worrisome in real-time systems because if there are errors or lags in the data processing, the quality of the generated responses can be considerably decreased [56]. For example, Kalman filters and smoothing algorithms are widely used in preprocessing to reduce noise, so that the data transferred to the AI model are of the required accuracy [57]. Also, real-time data validation is very important to crosscheck that only valid data affect the system, thereby improving the reliability of interactions [58].

In addition to the challenges posed by noisy data, handling large volumes of real-time sensor data requires significant computational resources. While models like ChatGPT are efficient, combining them with continuous sensor input demands robust processing infrastructure capable of managing a high throughput and minimizing latency. To meet these requirements, edge computing solutions or optimized data pipelines can be implemented, ensuring that real-time processing is both efficient and timely for sensor-driven AI applications [17,59].

Moreover, the use of sensor data, particularly when it include personal or sensitive biometric information, raises important privacy and security concerns. To protect user data, encryption protocols and secure transmission methods are essential, ensuring that data integrity is maintained throughout its journey to the AI system [60]. Transparent consent management is equally critical, with users needing a clear understanding of how their data are used in generating responses, thereby promoting informed participation and trust in sensor-driven AI systems [61].

Integrating real-time sensor data with large language models (LLMs) like ChatGPT offers promising advancements, but it also presents a host of technical and practical challenges. Since sensor data are often volatile and prone to fluctuations, maintaining its accuracy is a significant hurdle. This becomes even more critical in real-time systems, where any delays or errors in data processing can undermine the quality of AI-generated responses. To mitigate this, preprocessing techniques such as Kalman filters and smoothing algorithms are essential for noise reduction, ensuring that only reliable data are used as AI input. Additionally, real-time data validation is crucial to verify the integrity of the incoming data, enhancing the overall reliability of system interactions. Moreover, handling large streams of sensor data necessitates considerable computational power. To address this, edge computing and optimized data pipelines are employed to minimize latency and ensure that processing remains efficient. Alongside these technical solutions, privacy and security concerns arise, especially when dealing with personal or biometric data. Implementing robust encryption methods and secure transmission protocols is vital for safeguarding data integrity. Furthermore, transparent consent management is essential, allowing users to understand how their data will be used in generating responses, which fosters trust and promotes responsible interaction with sensor-driven AI systems.

## 8. Integration in Healthcare, Smart Homes, and Industrial Applications: Integrating AI Models with Sensor Data for Enhanced Interaction and Predictive Insights

Incorporating sensor data into AI models such as ChatGPT is revolutionizing industries by enabling instant, contextually relevant responses and advanced forecasting capabilities. This synergy highlights the pivotal role of the sensor industry in driving transformative technological advancements. By integrating sophisticated sensor capabilities with the conversational skills of AI, businesses are achieving unprecedented levels of customization, automation, and productivity. For instance, wearable sensors are reshaping healthcare by providing real-time patient monitoring that enhances diagnosis and treatment, showcasing the essential role of sensor technologies in improving care. In smart home environments, devices equipped with sensors combined with AI-powered assistants are streamlining household management, offering a seamless user experience. Similarly, industrial IoT applications are leveraging sensor data to enable predictive maintenance, reducing downtime and enhancing operational efficiency. As these examples demonstrate, the sensor industry is at the forefront of innovation, supplying the foundational technologies that empower AI-driven systems to deliver meaningful, real-world benefits. The expanding adoption of sensors in healthcare, smart home, and industrial settings underscores the growing demand for advanced, reliable sensor solutions, solidifying the industry’s critical role in shaping a smarter, more connected future.

### 8.1. Healthcare: Wearable Sensors and AI Chatbots for Patient Care

Wearable sensors have become a necessary element of modern healthcare, enabling continuous monitoring of vital signs, physical activity, and different health metrics. These sensors provide immediate updates on patients’ conditions, creating opportunities for proactive and personalized treatment [62]. Wearable sensors, when combined with AI chatbots like ChatGPT, have the ability to empower virtual assistants to deliver intelligent responses that are tailored to the specific context. For instance, wearable technology such as glucose monitors, ECG sensors, or continuous blood pressure monitors transmit patient data to an AI-powered virtual assistant, enabling tailored and immediate engagements depending on the patient’s health status [63]. One crucial application of this technology is in managing long-term health conditions such as diabetes or heart issues, where continuous supervision and immediate responses from AI systems can significantly improve disease management and patient outcomes [63,64].

A typical wearable device such as the Apple Watch or Fitbit tracks vital signs like the heart rate, blood oxygen levels, and steps, while more specialized devices (e.g., Dexcom G6 for diabetes) monitor glucose levels in real time. These devices sync their data to a cloud-based system that communicates with AI chatbots. The chatbot interprets the data from the wearable sensors and generates personalized feedback. Similarly, a diabetic patient using a continuous glucose monitor (CGM) can receive real-time notifications from a ChatGPT-powered virtual assistant. If the sensor detects an abnormal glucose level, the AI could provide immediate suggestions such as, “Your blood sugar level is higher than normal. Would you like some tips on how to manage it, or would you like me to contact your healthcare provider?”. In severe cases, the AI could escalate the issue by alerting medical personnel automatically. This integration offers real-time interventions and reduces the need for frequent doctor visits, providing continuous support. Furthermore, the conversational nature of the AI allows patients to ask follow-up questions, seek advice, and receive support in a more personalized manner, leading to better patient engagement and potentially improving health outcomes.

Combining wearable sensors with AI models can also aid in the treatment of mental health disorders like anxiety and depression. Wearable devices such as the Oura Ring and Whoop Strap monitor factors like sleep quality, HRV, and activity levels, which can show signs of stress or mental health problems. These devices gather information on the user’s physical and physiological condition, which is then transmitted to an artificial intelligence system. Virtual assistants powered by ChatGPT can analyze this information and interact with users in valuable discussions to assess their emotional health. Should the wearable sensor notice a decrease in HRV or irregularities in sleep patterns, the virtual assistant could comment, “It appears that your HRV has dropped in the past few days. Do you feel overwhelmed or uneasy? In what way can I help you deal with these emotions?”.

For example, a patient suffering from long-term anxiety might be provided with immediate updates on their stress levels or sleeping habits. If it seems like the stress levels are increasing based on the data, the virtual assistant may suggest stress-relief options such as mindfulness exercises or advise setting up a therapy appointment with a counselor. This enables users to obtain prompt, context-specific interventions using their live data. The combination of wearable sensors and AI chatbots not only assists users in gaining a deeper understanding of their physical and emotional health but also provides them with self-management tools. Ongoing observation allows for the timely identification of worsening conditions, which may help in averting more severe mental health episodes.

### 8.2. Smart Homes: AI-Assistant-Enhanced Interaction Through Sensors

Smart homes are technically the most advanced examples of sensor-driven, AI-powered systems that can monitor the physical and motion sensors that are used to make decisions based on the sensed data and the events in the home. These sensors can be integrated with AI helpers such as ChatGPT, which enables individuals to interact with their residences in a more seamless and effective manner. A key example of how AI can be utilized in the IoT to provide services within a smart home is energy management. Thermostats, lighting systems, and appliances that have sensors can work with AI assistants to improve energy efficiency by considering factors like occupancy, room temperature, and time of day [65,66]. Devices such as the Nest Thermostat and Ecobee build their functions around the environmental conditions using information from the sensors, which guarantees the home is energy-efficient automatically. These systems employ AI models like ChatGPT, which process data from sensors and make adjustments or decisions based on user preferences, environmental conditions, or external factors like the weather [67]. This harmony of updating, energy-saving impulsiveness offers further comfortability, which eliminates the necessity of manual changes and increases the possibility of energy consumption decreases, while providing a proactive, customized user experience.

### 8.3. AI for Home Security and Safety

The integration of AI technologies in smart home systems can enhance security and safety by merging motion detection, cameras, and environmental sensors such as smoke detectors and CO_2_ monitors to assess possible threats and send prompt alerts or responses. An instance includes the utilization of security systems like a Ring Doorbell and Nest Protect, which combine motion, sound, and camera sensors to monitor a house’s perimeter. When integrated with AI models like ChatGPT, these systems can analyze sensor information to detect potential safety concerns and respond accordingly. If a smoke detector detects elevated CO_2_ or smoke levels, ChatGPT might notify, “There could be a fire hazard in the kitchen. Would you like me to contact emergency services or assist you in creating a plan to evacuate the building?” Similarly, if movement is detected when the house is expected to be vacant, the system might ask, “It seems like there is activity detected in the living area. Would you rather have a live video stream or should I reach out to a security company?”. These AI-driven responses not only enhance security but also provide users with relevant information to efficiently deal with potential threats.

## 9. Ethical Considerations

As the integration of sensor technologies with AI models continues to advance, several ethical concerns warrant careful examination. One critical issue is the potential for privacy breaches. Sensor networks and IoT devices continuously collect sensitive, real-time data about individuals, including biometric information, environmental conditions, and user behaviors. When these data are combined with AI systems like ChatGPT, the risk of unauthorized access or misuse increases significantly. Without robust security protocols and clear regulatory frameworks, users may face serious risks such as data exploitation, identity theft, or unwarranted surveillance. Ensuring user trust in these technologies requires the development and enforcement of stringent data protection measures, such as encryption, access control, and transparency in data handling practices.

Another ethical challenge lies in the potential biases present in both sensor technologies and AI models. Sensors may inadvertently capture data that reflect systemic inequities, and AI systems trained on biased datasets can amplify these issues. For instance, in healthcare applications, wearable sensors integrated with ChatGPT could deliver recommendations that are less accurate or effective for specific demographic groups due to biases in the collected data or limitations of the sensors themselves. Such disparities could exacerbate existing inequalities, particularly for marginalized populations. Addressing this concern requires adopting inclusive design principles, conducting rigorous bias audits, and fostering accountability at every stage of system development and implementation.

Moreover, the algorithms used to process sensor data in AI models, such as ChatGPT, may inherit or exacerbate biases present in the data. For example, if sensor networks disproportionately collect data from certain geographic areas, socio-economic groups, or device users, AI predictions and recommendations may skew toward these groups, ignoring or misrepresenting others. Such biases can lead to discriminatory outcomes in areas like healthcare, housing, and employment, where AI-driven decisions have significant consequences. Mitigating these risks requires algorithmic transparency, continuous monitoring for bias, and the use of diverse datasets that accurately represent the populations being served.

Additionally, the widespread deployment of sensors raises societal concerns about surveillance and the erosion of privacy. Ubiquitous sensor networks in public and private spaces could normalize constant monitoring, leading to a surveillance society where individuals feel they must sacrifice personal freedoms for convenience or security. This could result in power imbalances, with those who control the data wielding disproportionate influence over society. Policymakers and technologists must work together to ensure that sensor deployment aligns with ethical standards, protecting individual rights while enabling technological progress.

## 10. Final Thoughts

Energy efficiency, scalability, and context-awareness are pivotal in optimizing sensor networks for diverse applications, from healthcare to smart homes [68,69]. Leveraging techniques like duty cycling and energy harvesting significantly extends the battery life of sensor nodes, while advanced communication protocols enhance sustainability. Simultaneously, scalable data aggregation and distributed processing frameworks enable real-time analysis, ensuring performance in vast sensor networks. Integrating sensor data with AI models like ChatGPT introduces a new dimension of interaction by generating context-sensitive dialogues tailored to user and environmental inputs. This capability transforms applications, offering personalized healthcare recommendations, adaptive smart home controls, and predictive maintenance in industrial settings. Despite challenges like noisy data, computational demands, and privacy concerns, advancements in preprocessing, data fusion, and secure frameworks promise robust solutions. By harnessing the synergy of sensor networks and AI, we can unlock transformative potential, driving innovation across industries while enhancing the user experience and operational efficiency.

## Figures and Tables

**Table 1:** Example flowchart.

A [Start] --> B (Acquire Raw Sensor Data)
B --> C {Data Preprocessing Needed?}
C -- Yes --> D (Normalize Sensor Values)
C -- No --> G (Fuse with Other Context)
D --> E (Create Contextual Embeddings)
E --> F (Structure Data for ChatGPT Input)
F --> G (Fuse with Other Context)
G --> H (Input Data to ChatGPT)
H --> I (Generate Context-Aware Dialogue)
I --> J [End]

## Data Availability

The original contributions presented in this study are included in the article. Further inquiries can be directed to the corresponding author.
